# Autoimmune Polyglandular Syndrome Type 1: A Report of Rare Case of a 16-Year-Old Asian Female Patient With Respiratory Manifestations

**DOI:** 10.7759/cureus.28741

**Published:** 2022-09-03

**Authors:** Kaneez Zainab, Mishaal Munir, Asfand Yar Cheema, Muhammad Hamza Qureshi, Pakeezah Bisharat

**Affiliations:** 1 Internal Medicine, Mayo Hospital, Lahore, PAK; 2 Medicine, Ghurki Trust Teaching Hospital, Lahore, PAK; 3 Medicine, Lahore Medical and Dental College, Lahore, PAK; 4 Medicine, Services Hospital Lahore (SHL), Lahore, PAK; 5 Internal Medicine, Lahore Medical and Dental College, Lahore, PAK; 6 Internal Medicine, Khyber Medical University, Peshawar, PAK

**Keywords:** generalized tonic-clonic seizures, immune dysregulation, pediatrics, auto-imune diseases, aps type i, keratitis, autoimmune adrenal insufficiency, hypoparathyroidism, mucocutaneous candidiasis

## Abstract

Autoimmune Polyglandular Syndrome (APS) spans three types of autoimmune disorders, categorized as APS type 1, type 2, and type 3. APS type 1 (APS-1) is the rarest type of the three. Complications of APS-1 can affect the bones, joints, skin, nails, gonads, eyes, thyroid, and several internal organs. We report a case of APS-1 in a 16-year-old female patient, who presented with complaints of oral thrush, tingling and numbness in her peripheries, and rash with multiple patches on the whole body with an infection of the nails, all around the age of 11 years, progressively worsening with time. In the next two years, she developed a bilateral decrease in visual acuity and dryness of the eyes as well as seizures, which have previously been seen in APS-1 patients. Laboratory results revealed hypocalcemia and hypoparathyroidism, but normal morning cortisol. Her mucocutaneous candidiasis and hypoparathyroidism fulfilled the criteria required for diagnosing APS-1. Her case, however, was made unique by her respiratory manifestations of frequent respiratory tract infections, which started around the age of 15 years, with a high-resolution CT scan (HRCT) revealing bronchiectasis, not commonly found amongst APS-1 patients. She was prescribed antibiotics for her respiratory tract infection exacerbations, oral calcium, vitamin D supplements, oral fluconazole, as well as an extensive eye care regimen. We report this case to add to the knowledge of this rare disease and look at its usual as well as unusual manifestations.

## Introduction

Autoimmune Polyglandular Syndrome (APS) is a group of disorders that include various clinical entities of immune dysregulation and systemic or hormonal deficiencies. APS type 1 (APS-1) is a rare, autosomal recessive inherited disorder encompassing chronic mucocutaneous candidiasis, hypoparathyroidism, and autoimmune adrenal insufficiency, with the presence of two of these being essential for diagnosis [1]. The pathogenesis of the syndrome involves the development of T cells with a high affinity to auto antigens; a defect in the body’s immune system fails to eliminate these T cells, and thus they bind to and attack the host’s cells. Most patients manifest the disorder as early as five years of age, while the complete presentation of the triad of disorders is usually completed in the first 20 years of life [2].

## Case presentation

We present the case of a 16-year-old Asian female patient with features suggestive of APS type 1. The young patient first complained of recurrent white patches in the mucosa of her mouth, rashes with multiple patches on the whole body, and tingling and numbness in her peripheries around the age of 11 years. She also complained of bilateral decreased visual acuity and dryness of the eyes, making it extremely difficult to open her eyes. Examination revealed a young female with a height in the fourth percentile and a thin body habitus with rashes on the arms and abdomen. An assessment concluded with the diagnosis of mucocutaneous candidiasis, tenia corporis, and blepharitis. The following year, she also reported having generalized tonic-clonic seizures. Furthermore, an electroencephalogram (EEG) revealed epileptiform activity with a generalized burst of sharp waves followed by slow waves. 

The constellation of findings prompted further investigation, which revealed a low hemoglobin of 9mg/dl, serum calcium of 4.4 mg/dl (normal 8.8-10.6 mg/dl), parathyroid hormone (PTH) 2.50 pg/ml (normal 12-88 pg/ml), morning cortisol 350 nmol/L (normal before 10 AM = 123-626 nmol/L). Serum sodium, potassium, magnesium, immunoglobulin E (IgE), and thyroid-stimulating hormone (TSH) were normal. Serum for anti-acetylcholine receptor and anti-transglutaminase antibodies were negative. An orthopantomogram revealed multiple impacted permanent teeth. Therefore, keeping in mind the multiple complaints, and laboratory and clinical evidence of disease, she was diagnosed with APS-1.

The patient returned three years later with fever, productive cough, which was bloody and foul-smelling, shortness of breath, and recurrent respiratory tract infections. Her oxygen saturation (SpO2) was 86% on room air; she had bilaterally reduced breath sounds with coarse crepitations. Skin examination revealed a diffuse rash over the arms and abdomen, characterized by multiple reddish ovoid lesions with patches and plaques. Furthermore, oral thrush, clubbing, and pallor were also present. The patient underwent high-resolution computed tomography (HRCT) scan of the chest, revealing bilateral ground-glass haze in a mosaic pattern in the bilateral upper lobes with central bronchiectasis. In addition, due to her ongoing complaints of epilepsy, we did a CT scan of the brain, which showed bilateral basal ganglia calcifications. 

The patient was treated symptomatically with oxygen inhalation, antibiotics including moxifloxacin 400mg IV once daily, piperacillin-tazobactam 3.375 g IV four times a day, oral fluconazole 200mg once daily, and topical terbinafine. Oral valproic acid 500mg twice daily, as prescribed earlier for epilepsy was continued. An extensive eye care regimen was prescribed, including eye lubricants and regular cleansing of the eyes. In addition, we optimized iron, calcium, and vitamin D supplementation. Her test reports returned to her previous baseline results, which was 8.5mg/dl for calcium, and hence she was discharged with a plan for regular follow-up.

## Discussion

APS-1 is a rare disorder that primarily affects young patients, both males and females equally, and manifests as chronic mucocutaneous candidiasis, hypoparathyroidism, and Addison disease. However, further manifestations include chronic diarrhea, teeth abnormalities, and primary ovarian insufficiency [3]. The pathogenesis involves the loss of function mutations in the autoimmune regulator gene on chromosome 22q22.3, which is essential for the auto apoptosis of T cells [4]. The mutation leads to the development of T cells that attack the own body's tissues and thus cause the manifestations of APS-1.

The patient in the case above is a rare presentation of a sporadic disorder, with respiratory manifestations being a very unusual finding. She had an early onset of recurrent mucocutaneous candidiasis, followed by blepharitis, symptoms of hypocalcemia, and seizures. While the presentation of APS-1 is usually in chronological order, with candidiasis presenting around five years of age, followed by hypoparathyroidism around 10 years, and then Addison's disease around 15 years, this specific order occurs in only one-third of the population [5]. In addition, keratitis has been seen in one-fourth of patients with the disease, manifesting as dryness of the eyes, photophobia, and blepharospasm [6]. Thus a high clinical index of suspicion should be kept even in the absence of the classic features. In our patient, the recurrent episodes of mucocutaneous candidiasis presented as rashes on the body, onychomycosis, and white patches of thrush in the mouth.

Our patient's several laboratory tests revealed low serum calcium of 4.4mg/dl, associated with symptomatic hypocalcemia. The intact parathyroid hormone (PTH) was also low, while the rest of her screen, including serum cortisol and thyroid profile, were unremarkable. Hypoparathyroidism is one of the cardinal features of APS-1; when an endocrine disorder is the first presenting disorder, it is almost invariably hypoparathyroidism [2]. It is often diagnosed when symptoms of hypocalcemia develop. The third feature of APS-1, not present in our patient, is adrenal insufficiency. Irvine and Barnes have reported a bimodal age distribution at autoimmune adrenalitis onset; the first peak occurs from 10 to 15 years, while a second peak, later on, can occur in the fifties, affecting mainly women with Type 1 diabetes, autoimmune thyroid disease, or both, presenting in about 84% of APS-1 patients [7]. Keeping that in mind, it might be possible that she had not as yet developed Addison's disease and would develop it as the disease progresses.

The nails and teeth are most likely to be affected in 80% of individuals, having non-endocrine features [4]. In addition, enamel defects, including hypoplastic and hypo-mineralized enamel, can present as an early sign of APS-1 and aid in diagnosing the disorder [8]. Our patient had both manifestations, as she had onychomycosis and teeth malformations.

**Figure 1:**
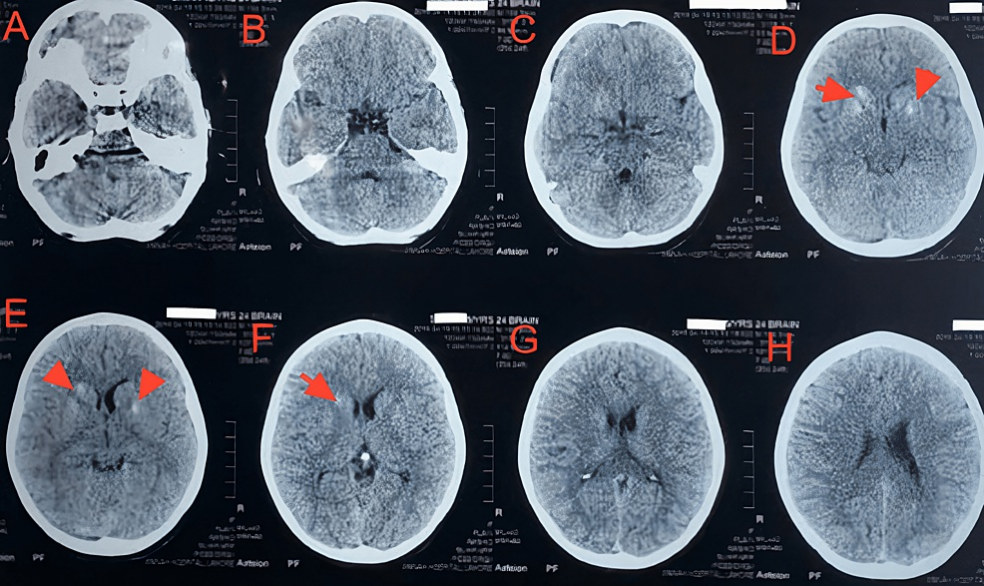
Plain CT brain, axial sections The red arrows in sections D, E, and F depict basal ganglia calcifications, which constitute Fahr's syndrome, present in patients with hypoparathyroidism and APS-1 APS-1: Autoimmune Polyglandular Syndrome type 1

When reviewing neurological manifestations of the syndrome, seizures have been reported occasionally in patients with APS-1 [9]. Our patient reported generalized tonic-clonic seizures, with EEG showing epileptiform activity. Further imaging of the brain showed basal calcifications, as seen in Figure 1, which, although seen in advanced age, are considered abnormal in a young female.

Evidence of basal ganglia calcification, known as Fahr's syndrome, has been seen in specific genetic mutations and disorders of calcium/phosphorus homeostasis, especially hypoparathyroidism [10]. Fahr's syndrome explains the basal ganglia calcifications in our patient, prompted by hypoparathyroidism, paired with the seizures she experienced.

**Figure 2:**
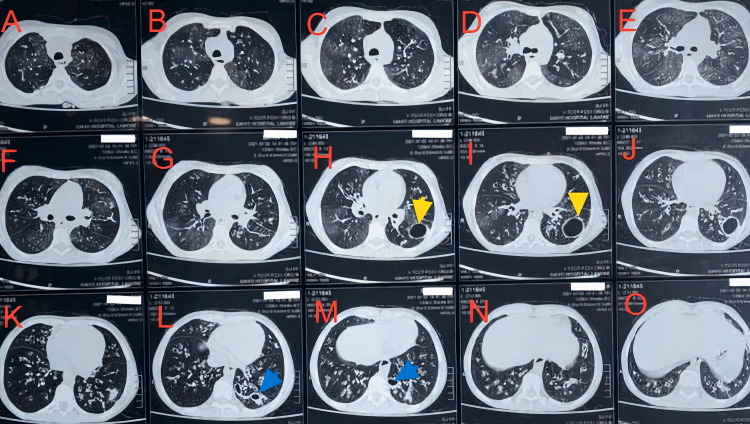
HRCT chest, axial sections Sections A to H show diffuse mosaic attenuation, the yellow arrows in sections H and I point to a cavity formed from repeated infections, and the blue arrows in sections L and M show signet ring sign, which depict bronchiectasis HRCT: high-resolution computed tomography

However, the most interesting aspect of our patient's presentation was her respiratory tract abnormalities. Not usually included in the spectrum of disorders that APS-1 patients develop, she experienced recurrent chest infections, around two to three episodes per year, with a drop in her SpO2 to 86% on room air. An HRCT chest, as shown in Figure 2, depicted evidence of bronchiectasis and a cavity formation as a result of recurrent infections, while her echocardiogram was normal. A few cases have been previously identified, with evidence of autoimmune pneumonitis, clinically defined by respiratory function symptoms lasting more than four weeks, ranging from interstitial lung disease to bronchiectasis [11]. They may have an obstructive, restrictive or mixed pattern. Our patient started coughing with sputum production around 12 years of age and presented with oxygen dependency by 16. A similar report has reported a patient being oxygen dependent by 19 years of age and another dying of respiratory failure at 37 years of age [11]. The quick progression in our patient might have been contributed to by a lack of access to medical facilities; by the time she presented to us, her pulmonary function had mainly been compromised. 

Apart from the manifestations listed above, APS-1 patients can also develop hypogonadism, vitiligo, chronic diarrhea and gastritis, autoimmune hepatitis, autoimmune hypothyroidism, type 1 diabetes mellitus, and pituitary failure [6].

## Conclusions

Like most other autoimmune conditions, APS-1 manifests in early life and affects several systems of the body, including the eye, endocrine, nervous and immune systems as well as the skin. Patients are vulnerable to electrolyte abnormalities, infections, development of seizures, and visual abnormalities. As respiratory manifestations in this rare disease are very rare, our case adds greatly to the existing literature pertaining to it. Further research is required to decide whether genetic, environmental, or other factors play a role in affecting the lungs. However, it is evident from our case that the manifestations of APS-1 proliferate very quickly, greatly deteriorating the quality of life, thus multi-specialty involvement is required very early on to ensure that the patient has a better prognosis and management.
